# Supplementary material for: The causal role of circulating inflammatory markers in osteoporosis: a bidirectional Mendelian randomized study
Source: Front Immunol. 2024 Jul 18;15:1412298. doi: 10.3389/fimmu.2024.1412298 (PMC11291241; doi:10.3389/fimmu.2024.1412298)
Supplement: Supplementary file 1 [file DataSheet_1.zip › supplementary material/OSTEOPOROSIS-CIM/GCST90274823.tsv.gz_leave_one_out.pdf]

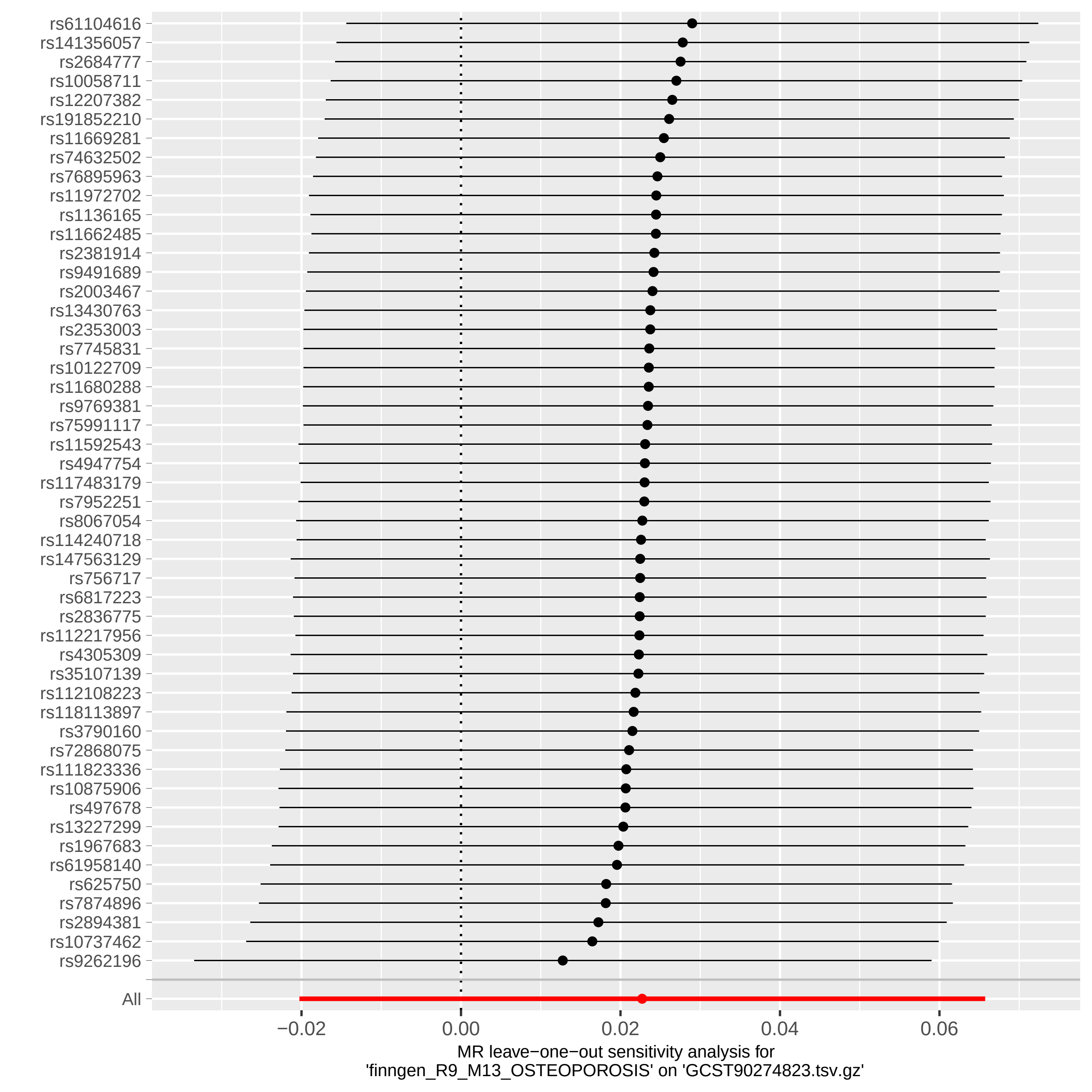

rs61104616
rs141356057
rs2684777
rs10058711
rs12207382
rs191852210
rs11669281
rs74632502
rs76895963
rs11972702
rs1136165
rs11662485
rs2381914
rs9491689
rs2003467
rs13430763
rs2353003
rs7745831
rs10122709
rs11680288
rs9769381
rs75991117
rs11592543
rs4947754
rs117483179
rs7952251
rs8067054
rs114240718
rs147563129
rs756717
rs6817223
rs2836775
rs112217956
rs4305309
rs35107139
rs112108223
rs118113897
rs3790160
rs72868075
rs111823336
rs10875906
rs497678
rs13227299
rs1967683
rs61958140
rs625750
rs7874896
rs2894381
rs10737462
rs9262196
All
−0.02
0.00
0.02
0.04
0.06
MR leave−one−out sensitivity analysis for
'finngen_R9_M13_OSTEOPOROSIS' on 'GCST90274823.tsv.gz'
